# A new genus, two new species and a new record of subfamily Cecidophyinae (Acari, Eriophyidae) from China

**DOI:** 10.3897/zookeys.180.2641

**Published:** 2012-04-05

**Authors:** Guo-Quan Wang, Sui-Gai Wei, Ding Yang

**Affiliations:** 1 Department of Plant Protection, Agricultural College, Guangxi University, Nanning, China

**Keywords:** Eriophyoidea, eriophyoid mites, Cecidophyini, Colomerini, taxonomy, China

## Abstract

A new genus and two new species belonging to subfamily Cecidophyinae, namely *Kyllocarus reticulatus* **gen.n., sp. n.** infesting *Lithocarpus brevicaudatus* (Skan) Hay. (Fagaceae) and *Gammaphytoptus schimae* **sp. n**.infesting *Schima superba* Gardn. et Champ. (Theaceae) are described and illustrated. Both new species are vagrants on their respective host plants. *Cecidophyes digephyrus*Keifer, 1966 is newly recorded for China.

## Introduction

The subfamily Cecidophyinae holds two tribes, Cecidophyini and Colomerini, which were differentiated by the former scapular tubercles and setae are absent and the later tubercles and setae are present. So far, sixteen genera and thirty-three species are known from China (Huang 2001; Huang and Cheng 2005; Huang and Wang 2004, 2009; Kuang 1987, 1995, 1997, 1998; Kuang and Hong 1992, 1995; Kuang and Luo 1992; Kuang, Luo and Wang 2005; Li et al. 2009; Song et al. 2008; Wang et al. 2009; Wei et al. 2009; Xue et al. 2009). Herein, one new genus and new species *Kyllocarus reticulatus* gen. n., sp. n., in the tribe Cecidophyini and another new species, *Gammaphytoptus schimae*sp. n., in the tribe Colomerini are described and illustrated.

## Materials and methods

Specimens were located with the aid of a magnifying glass on plant material in the field, and specimens were collected into and preserved in a sucrose-ethanol solution (75%). The mites were cleared in Nesbitt’s solution and mounted in Heinze medium on glass slides at room temperature according to Kuang (1986). Specimens were measured follows de Lillo et al. (2010). The morphological terminology and the generic classification follow Amrine et al. (2003).

Type specimens are deposited in the Department of Plant Protection, Guangxi University, Nanning. All measurement units are in micrometers (*μ*m) and rounded off to the nearest full number, and are lengths when not specified. Specimens were examined with an Olympus CX41 (Philippines) microscope with phase contrast. The number of measured specimens is given in parentheses.

## Taxonomy

### Tribe Cecidophyini Keifer, 1966. Genus Cecidophyes Nalepa, 1887

**Type species.** *Phytopus galii* Karpelles, 1884.

#### Cecidophyes digephyrus

Keifer, 1966 rec. n.

http://species-id.net/wiki/Cecidophyes_digephyrus

##### Diagnosis.

Body fusiform. Prodorsal shield with frontal lobe present; median, admedian lines and submedian lines complete, connected with three transverse lines forming network; scapular tubercles and setae absent. Coxisternal plates sculptured with lines. Legs with normal segments and usual setae, tarsal empodium entire 6-rayed, tarsal solenidion knobbed. Dorsal opisthosoma evenly rounded, dorsal annuli 62–68, with elongated microtubercles; ventral annuli 62–68, with rounded microtubercles, setae *h1* absent. Female genital near coxisternal plates, coverflap with two rows of ridges.

##### Material examination.

10 females, Qingliangfeng National Nature Reserve, Lin’an City (30°10'N, 119°07'E), Zhejiang Province, China, 24. VII. 2007, from *Quercus* sp. (Fagaceae), collected by Guo-Quan Wang, mounted on individual slide.

##### Distribution.

USA, China (Zhejiang).

##### Relation to host.

The mites are vagrants on the surfaces of the leaves, no visible damage seen.

##### Remarks.

Up to date, nine species of *Cecidophyes* have been known infesting *Quercus* spp.: *Cecidophyes caliquerci* (Keifer, 1944) infesting *Quercus lobata* Nee and *Quercus marilandica* Muen., *Cecidophyes digephyrus* Keifer, 1966 infesting *Quercus vaccinifolia* Kell., *Cecidophyes lyrata* Keifer, 1959 infesting *Quercus lyrata* Walt., *Cecidophyes pusilla* Keifer, 1962 infesting *Quercus falcata* Michx, *Cecidophyes quercialbae* Keifer, 1959 infesting *Quercus alba* L., *Cecidophyes querciphagus* (Keifer, 1939) infesting *Quercus* sp., *Cecidophyes reticulatus* Livshitz, Mitrofanov *et* Vasil’yeva, 1979 infesting *Quercus pubescens* Willd.*, C. tampae* Keifer, 1966 infesting *Quercus virginiana* and *Cecidophyes tristernalis* (Nalepa, 1898) infesting *Quercus cerris* L. Among them, only one species, *Cecidophyes tampae* occurred in China. *Cecidophyes digephyrus* is second *Cecidophyes* species from China (Keifer 1939, 1944, 1959a, b, 1962, 1966; Livshitz et al. 1979; Nalepa 1898; Xue et al. 2009).

#### Genus Kyllocarus

Wang, Wei & Yang, 2012 gen. n.

urn:lsid:zoobank.org:act:91681721-9DF7-483B-941C-C882DBF8A4FF

http://species-id.net/wiki/Kyllocarus

##### Type species:

*Kyllocarus reticulatus* Wang, Wei & Yang, 2012, sp. n.

The new genus with flattened fusiform body, palp genual seta strongly angular prodorsal shield lacking scapular setae (sc) and tubercles, strong wide frontal lobe over gnathosoma, legs normal, except leg II lacking genual seta *l’’*. Sternal apodeme present; opisthosoma differentiated into broader smooth dorsal annuli and narrower microtuberculate ventral annuli; empodium simple; genitalia very close to coxae, bearing two ranks of numerous ridges.

##### Diagnosis.

This cecidophyine mite is very near to *Kolacarus* in that the genual seta *l’’* is absent on Leg II; however, it differs from other cecidophine mites in that palp genual seta *d* is bent or crooked, possibly minutely bifurcate. It differs from *Kolacarus* in that the mite has a wide, strong frontal lobe projecting from the prodorsal shield over the gnathosoma; *Kolacarus* has a normal palp genual seta *d*, and no frontal lobe on the anterior prodorsal shield.

##### Etymology.

Kyllo- from Gr. Kyllos, crooked + -carus from Acarus; the name is masculine.

#### Kyllocarus reticulatus sp. n.

urn:lsid:zoobank.org:act:7358B76E-13F4-4968-82F4-2500479953D0

http://species-id.net/wiki/Kyllocarus_reticulatus

Figs 1–7

##### Diagnosis.

Body fusiform, white translucency or yellow. Gnathosoma curved obliquely downward, dorsal genual setae (*d*) bend forming obtuse angle at middle. Prodorsal shield with frontal lobe present; all lines bold and connected with transverse lines forming network; scapular tubercles and setae absent. Coxisternal plates sculptured with lines, prosternal apodeme present, coxigenital annuli 4. Legs segments normal, legs II with genual setae (*l’’*) absent, tarsal empodium entire, 6-rayed, tarsal solenidion knobbed. Dorsal opisthosoma with shallow median furrow, dorsal annuli smooth; ventral annuli with rounded microtubercles, setae *h1* absent. Female genitalia coverflap with two rows of ridges

##### Description.

Female (n = 11). Body fusiform, white translucency or yellow, 172 (150–204), 75 (69–79) wide, 60 (54–63) thick.

Gnathosoma. Curved obliquely downward, 30 (28–31), coxal setae (*ep*) 6 (6–7), dorsal genual setae (*d*) bend forming obtuse angle at middle, 11 (10–12); cheliceral stylets 31 (30–33).

Prodorsal shield. 63 (58–70), 69 (65–74) wide, frontal lobe present; all lines bold; median, admedian and submedian lines complete, connected with transverse lines forming network; scapular tubercles and setae absent.

Coxisternal plates. Prosternal apodeme present, coxisternal plates I and II sculptured with lines; anterolateral setae on coxisternum I (*1b*) 3 (3–4), 13 (12–13) apart; proximal setae on coxisternum I (*1a*) 5 (5–6), 13 (12–13) apart; proximal setae on coxisternum II (*2a*) 31 (29–33), 29 (29–30) apart. Coxigenital annuli 4.

Legs. Segments normal. Legs I 34 (30–37), trochanter 2 (2), femur 11 (10–11), femoral setae (*bv*) 13 (10–15); genu 5 (4–5), genual setae (*l’’*) 30 (29–32); tibia 7 (6–8), tibial setae (*l'*) located laterally and distally, 15 (13–18); tarsus 8 (7–9), inner fastigial tarsal setae (*ft’*) 27(25–28), outer fastigial tarsal setae (*ft’’*) 18 (15–20), unguinal tarsal setae (*u’*) 5 (4–5); tarsal empodium entire, 12 (11–13), 6-rayed, tarsal solenidion 7 (6–8), knobbed. Legs II 27 (26–30), trochanter 2 (2), femur 10 (10–11), femoral setae (*bv*) 23 (20–25); genu 4 (4–5), genual setae (*l’’*) absent; tibia 4 (4–5); tarsus 7 (6–7), inner fastigial tarsal setae (*ft’*) 24(23–25), outer fastigial tarsal setae (*ft’’*) 14 (13–15), unguinal tarsal setae (*u’*) 4 (4–5); tarsal empodium entire, 6 (5–7), 6-rayed, tarsal solenidion 7 (7–8), knobbed.

Opisthosoma. Dorsum with shallow median furrow, dorsal annuli 43, smooth; ventral annuli 63, with rounded microtubercles; setae *c2* 23 (20–25), on ventral annulus 10th; setae *d* 71 (63–79), 42 (41–43) apart, on ventral annulus 22th; setae *e* 11 (8–13), 24 (23–25) apart, on ventral annulus 38th; setae *f* 24 (22–25), 24 (24–25) apart, on 10th ventral annulus from rear; setae *h1* absent, setae *h2* 31 (26–39).

Female genitalia. Near coxisternal plates, coverflap with two rows of ridges, 24 (23–25), 43 (38–49) wide, proximal setae on coxisternum III (*3a*) 9 (9–10), 23 (23–24) apart.

Male (n = 2). Body fusiform, 120–140, 58–61 wide.

Prodorsal shield. 53–55, 55–57 wide, frontal lobe present; all lines bold; median, admedian lines and submedian lines complete, connected with transverse lines forming network; scapular tubercles and setae absent.

Coxisternal plates. Prosternal apodeme present, coxisternal plates I and II sculptured with lines; anterolateral setae on coxisternum I (*1b*) 3, 11 apart; proximal setae on coxisternum I (*1a*) 5, 11 apart; proximal setae on coxisternum II (*2a*) 27, 28 apart. Coxigenital annuli 4.

Legs. Segments normal. Legs I 30, trochanter 2, femur 10, femoral setae (*bv*) 12; genu 4, genual setae (*l’’*) 27; tibia 6, tibial setae (*l'*) located laterally and distally, 12; tarsus 7, inner fastigial tarsal setae (*ft’*) 24, outer fastigial tarsal setae (*ft’’*) 15, unguinal tarsal setae (*u’*) 4; tarsal empodium entire, 10, 6-rayed, tarsal solenidion 6, knobbed. Legs II 26, trochanter 2, femur 10, femoral setae (*bv*) 18; genu 4, genual setae (*l’’*) absent; tibia 4; tarsus 6, iner fastigial tarsal setae (*ft’*) 21, outer fastigial tarsal setae (*ft’’*) 12, unguinal tarsal setae (*u’*) 4; tarsal empodium entire, 5, 6-rayed, tarsal solenidion 7, knobbed.

Opisthosoma. Dorsum with shallow median furrow, dorsal annuli 42, smooth; ventral annuli 62, with rounded microtubercles; setae *c2* 20, on ventral annulus 10th; setae *d* 57, 40 apart, on ventral annulus 22th; setae *e* 7, 21 apart, on ventral annulus 38th; setae *f* 20, 21 apart, on 10th ventral annulus from rear; setae *h1* absent, setae *h2* 27.

Male genitalia. Near coxisternal plates, 36 wide, proximal setae on coxisternum III (*3a*) 8, 23 apart.

**Type material.** Holotype female, China: Zhejiang, Longquan City, Fengyangshan National Nature Reserve (27°53'N, 119°11'E), 27. VII. 2007, collected by Guo-Quan Wang, from *Lithocarpus brevicaudatus* (Skan) Hayata (Fagaceae). Paratypes, 10 females and 2 males.

**Figures 1–7.**
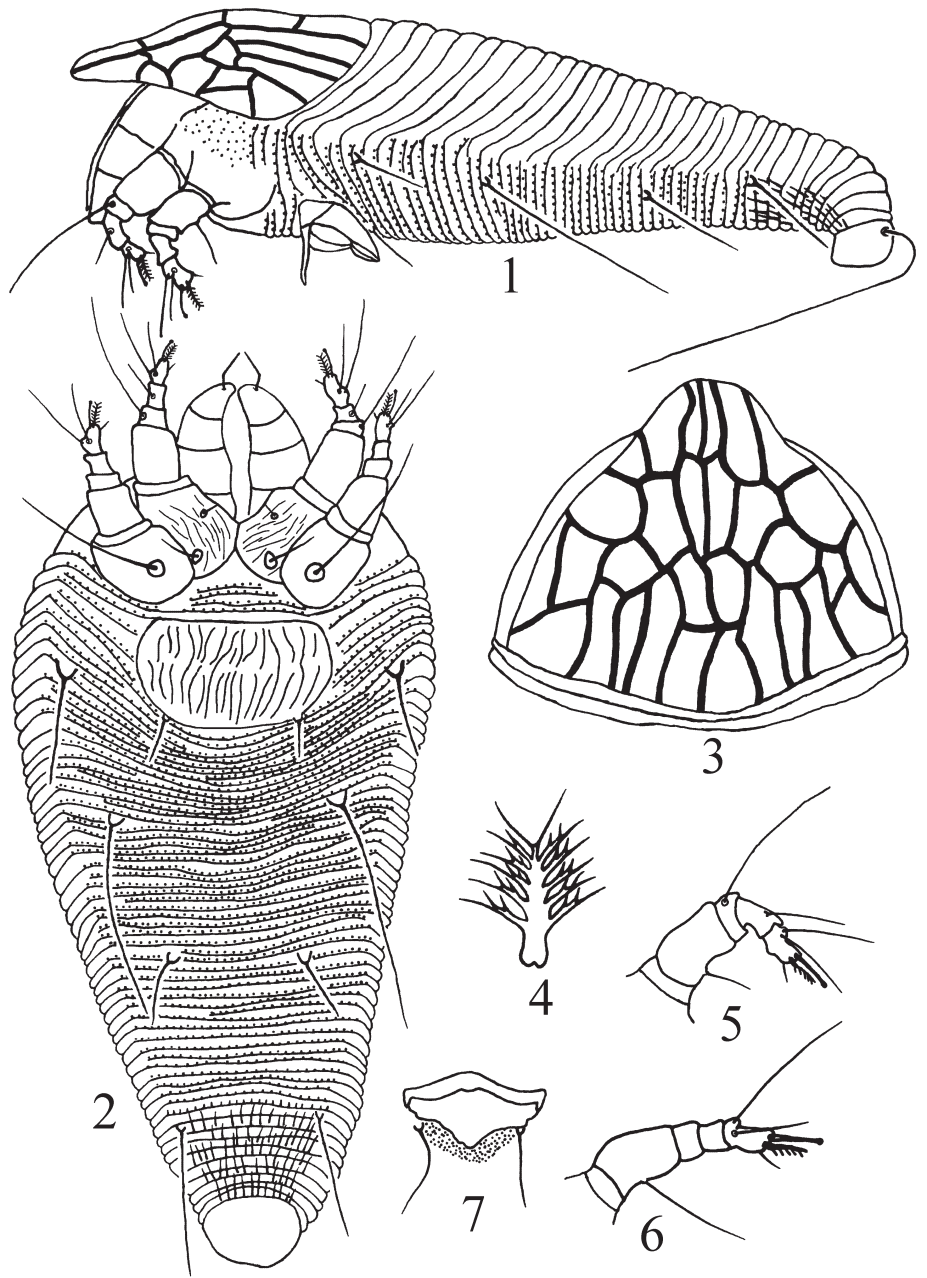
*Kolacarus reticulatus* sp. n. **1** lateral view of female **2** ventral view of female **3** anterior dorsal view of female **4** tarsal empodium **5** leg I **6** leg II **7** male genitalia

##### Distribution.

China (Zhejiang).

##### Etymology.

The species is named after the network-form of the prodorsal shield.

### Tribe Colomerini Newkirk & Keifer, 1975. Genus Gammaphytoptus Keifer, 1939

**Type species:** *Gammaphytoptus camphorae* Keifer, 1939.

#### Gammaphytoptus schimae sp. n.

urn:lsid:zoobank.org:act:CEC41DF7-A6BB-4871-9D06-FABC3DDAC219

http://species-id.net/wiki/Gammaphytoptus_schimae

Figs 8–13

##### Diagnosis.

Body fusiform, yellow. Gnathosoma curved obliquely downward, dorsal genual setae (*d*) bend forming obtuse angle at middle. Prodorsal shield with frontal lobe present; all lines bold and connected with transverse lines forming network; scapular tubercles and setae absent. Coxisternal plates sculptured with lines, prosternal apodeme present, coxigenital annuli 4. Legs segments normal, legs II with genual setae (*l’’*) absent, tarsal empodium entire, 6-rayed, tarsal solenidion knobbed. Dorsal opisthosoma with shallow median furrow, dorsal annuli smooth; ventral annuli with rounded microtubercles, setae *h1* absent. Female genitalia coverflap with two rows of ridges.

##### Description.

Female (n = 11). Body fusiform, yellow, 183 (169–200), 71 (65–78) wide, 44 (38–52) thick.

Gnathosoma. Curved obliquely downward, 34 (28–35), coxal setae (*ep*) 2 (2–3), dorsal genual setae (*d*) 10 (9–11); cheliceral stylets 30 (28–32).

Prodorsal shield. 51 (48–52), 55 (50–63) wide, frontal lobe present; median, admedian and submedian lines complete, connected with three transverse lines forming network; scapular tubercles placed at rear shield margin, 35 (31–39) apart, scapular setae (*sc*) 8 (8–9), directed backward and divergence.

Coxisternal plates. Prosternal apodeme present, coxisternal plates smooth; anterolateral setae on coxisternum I (*1b*) 8 (7–9), 13 (12–13) apart; proximal setae on coxisternum I (*1a*) 25 (19–31), 15 (14–15) apart; proximal setae on coxisternum II (*2a*) 35 (28–39), 28 (27–30) apart. Coxigenital annuli 4.

Legs. Segments normal. Legs I 36 (34–38), trochanter 2 (2), femur 12 (12–13), femoral setae (*bv*) 18 (15–22); genu 4 (4–5), genual setae (*l’’*) 35 (31–40); tibia 10 (9–10), tibial setae (*l'*) located 1/4 from apical, 8 (7–8); tarsus 8 (7–8), iner fastigial tarsal setae (*ft’*) 20 (18–23), outer fastigial tarsal setae (*ft’’*) 25 (23–28), unguinal tarsal setae (*u’*) 5 (5–6); tarsal empodium entire, 7 (7–8), 6-rayed, tarsal solenidion 10 (9–10), knobbed. Legs II 31 (29–34), trochanter 2 (2), femur 11 (11–12), femoral setae (*bv*) 23 (19–27); genu 3 (3–4), genual setae (*l’’*) 10 (7–12); tibia 7 (7–8); tarsus 8 (7–8), inner fastigial tarsal setae (*ft’*) 10 (9–12), outer fastigial tarsal setae (*ft’’*) 25 (22–29), unguinal tarsal setae (*u’*) 5 (5–6); tarsal empodium entire, 8 (8–9), 6-rayed, tarsal solenidion 10 (9–11), knobbed.

Opisthosoma. Dorsum evenly rounded, dorsal annuli 59–60, with semi-translucency elongated microtubercles; ventral annuli 81, with filament microtubercles; setae *c2* 38 (35–40), on ventral annulus 13th; setae *d* 45 (37–50), 43 (38–45) apart, on ventral annulus 28th; setae *e* 27 (23–32), 25 (23–26) apart, on ventral annulus 44th; setae *f* 38 (34–45), 23 (21–26) apart, on 7th ventral annulus from rear; setae *h1* absent, setae *h2* 57 (53–65).

Female genitalia. Near coxisternal plates, coverflap with two rows of ridges, 17 (16–18), 30 (29–22) wide, proximal setae on coxisternum III (*3a*) 20 (17–25), 13 (13–14) apart.

Male. Unknown.

**Figures 8–13.**
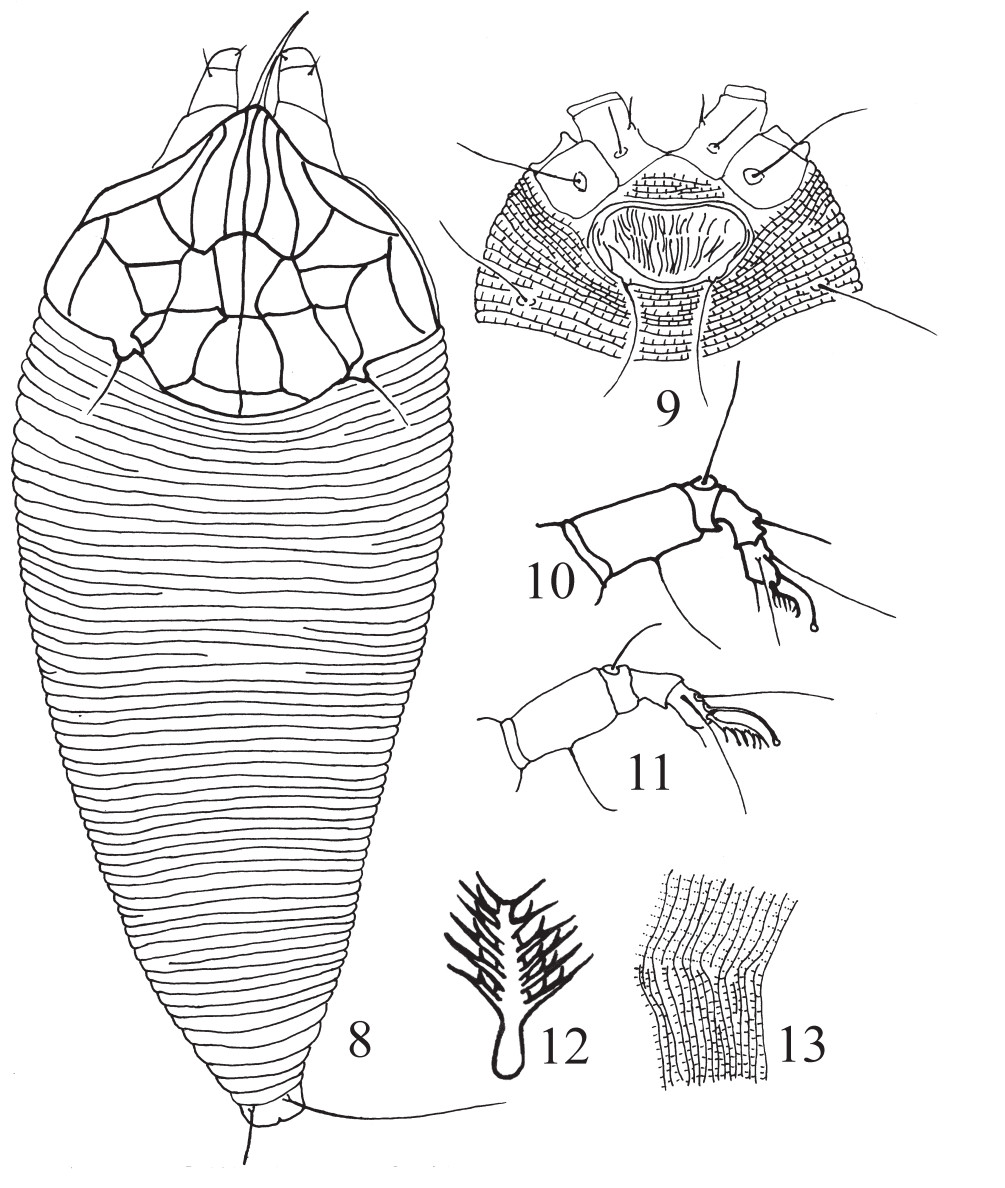
*Gammaphytoptus schimae* sp. n. **8** dorsal view of female **9** coxigenital area of female **10** leg I **11** leg II **12** tarsal empodium **13** lateral view of annuli.

##### Type material.

Holotype female, China: Zhejiang, Longquan City, Fengyangshan National Nature Reserve (27°53'N, 119°11'E), 28. VII. 2007, collected by Guo-Quan Wang, from *Schima superba* Gardn.&Champ. (Theaceae). Paratypes, 8 females.

##### Distribution.

China (Zhejiang).

##### Etymology.

The species is named from the generic name of the type host plant.

##### Remarks.

This new species is similar to *Gammaphytoptus zuihoensus* Huang & Wang, 2004, but they can be easily separated as follows: in *Gammaphytoptus schimae*, median line is complete, setae *h1* is absent and infesting *Schima superba* Gardn.&Champ.; in *Gammaphytoptus zuihoensus*, median line is incomplete, setae *h1* is present and infesting *Machilus zuihoensis* Hay. var. *zuihoensis* (Huang and Wang 2004).

## Supplementary Material

XML Treatment for Cecidophyes digephyrus

XML Treatment for Kyllocarus

XML Treatment for Kyllocarus reticulatus

XML Treatment for Gammaphytoptus schimae
